# Chondrosarcoma of Iliac Bone Imaging Spectrum With Histopathological Correlation: A Case Report

**DOI:** 10.7759/cureus.45110

**Published:** 2023-09-12

**Authors:** Suchita Bahurupe, Suresh Phatak, Prashant Onkar, Ashish N Ambhore, Deepali Trimukhe

**Affiliations:** 1 Radiodiagnosis, N. K. P. Salve Institute of Medical Sciences & Research Centre And Lata Mangeshkar Hospital, Nagpur, IND

**Keywords:** magnetic resonance imaging, computed tomography, ring and arc calcification, bone tumor, chondrosarcoma

## Abstract

Chondrosarcoma is a rare form of carcinoma that originates in the cells of cartilage, the flexible tissue that cushions the joints and gives structure to various parts of the body. This malignant tumour primarily affects adults and is most commonly found in the bones of the arms, legs, pelvis, and ribs. The severity and prognosis of chondrosarcoma can vary widely depending on factors such as tumour size, location, and grade. We are reporting a case of an 83-year-old male patient who presented with swelling over the left hip joint. A mass was detected on radiograph and ultrasound that was further characterized using contrast-enhanced CT and MRI. Imaging findings suggested chondrosarcoma. The patient was diagnosed on histopathological examination.

## Introduction

Chondrosarcomas comprise around 10-15% of malignant bone tumours, exhibiting slow growth and a higher occurrence in regions like the femur, humerus, pelvis, scapula, and ribs [1]. More uncommon sites encompass the neck and craniofacial area. Within bone locations, chondrosarcomas are typically categorized into two groups: those originating from the central or medullary cavity and those emerging from the bone's surface, termed peripheral or juxtacortical chondrosarcomas. While the majority of chondrosarcomas originate centrally and are primary, some can be secondary, stemming from an osteochondroma or enchondroma. These tumours predominantly manifest in adulthood or later stages of life, with a higher incidence in males. Clinical presentations commonly involve pain and swelling. Pathological classification of chondrosarcomas includes conventional and nonconventional subtypes, such as dedifferentiated, mesenchymal, clear cell, and extraskeletal myxoid variations [1].

## Case presentation

An 83-year-old male patient presented with swelling over the left hip and restricted joint movement in the last six months. The swelling was associated with pain. There was no history of trauma. On local examination, the swelling was soft, non-tender, and firm in consistency measuring 10 x 14 cm. No local rise in temperature or discolouration was noted.

**Figure 1:**
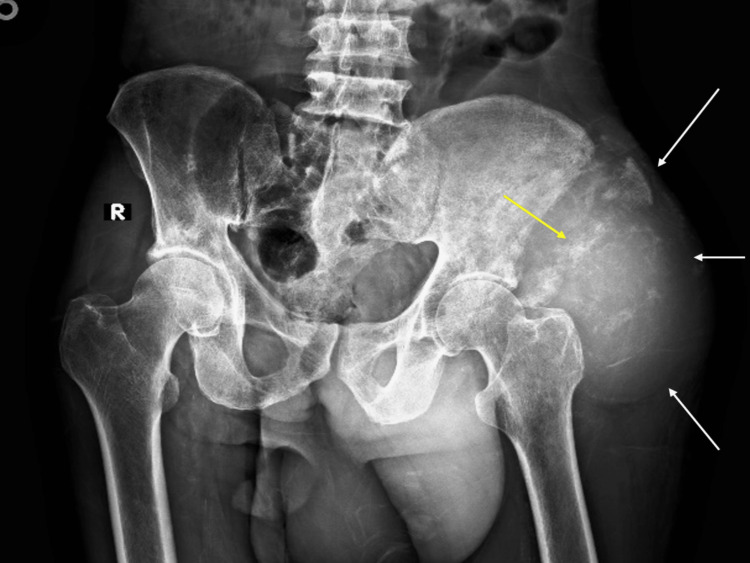
An ill-defined lobulated mass (white arrows) with calcific foci (yellow arrow) on left side causing displacement of left hip joint towards medial side.

On plain radiograph, an ill-defined, large lobulated soft tissue density lesion with permeative bone destruction was seen. Calcific foci were noted within it giving a heterogenous appearance suggestive of matrix mineralisation (Figure 1).

**Figure 2:**
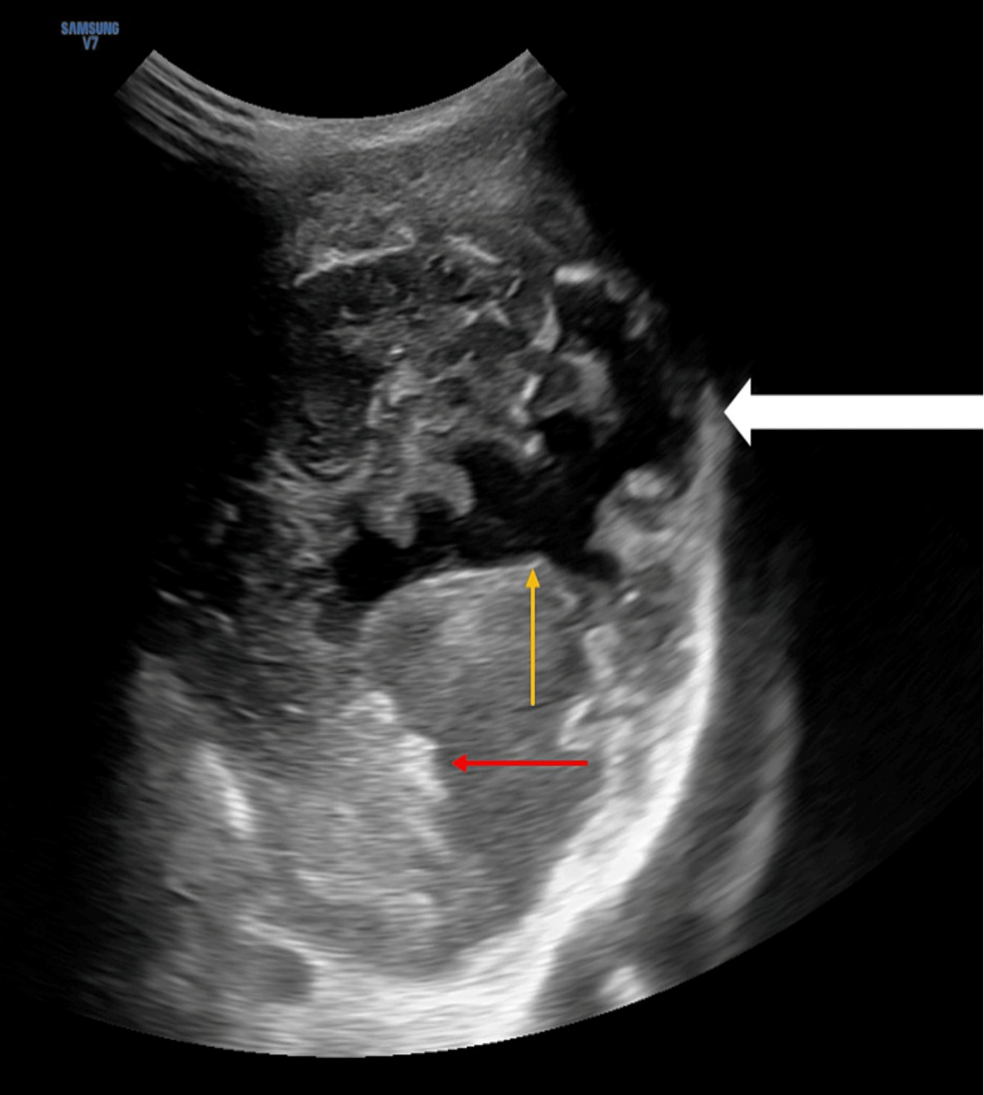
A large heterogeneous mass (white arrow) with flecks of calcification (red arrow) and cystic spaces (yellow arrow) noted within.

On high-frequency ultrasound, a large heterogeneous lesion with flex of multiple calcific foci was noted in the left iliac region. Some cystic changes were noted within it. The lesion showed no vascularity on colour Doppler (Figure 2).

**Figure 3:**
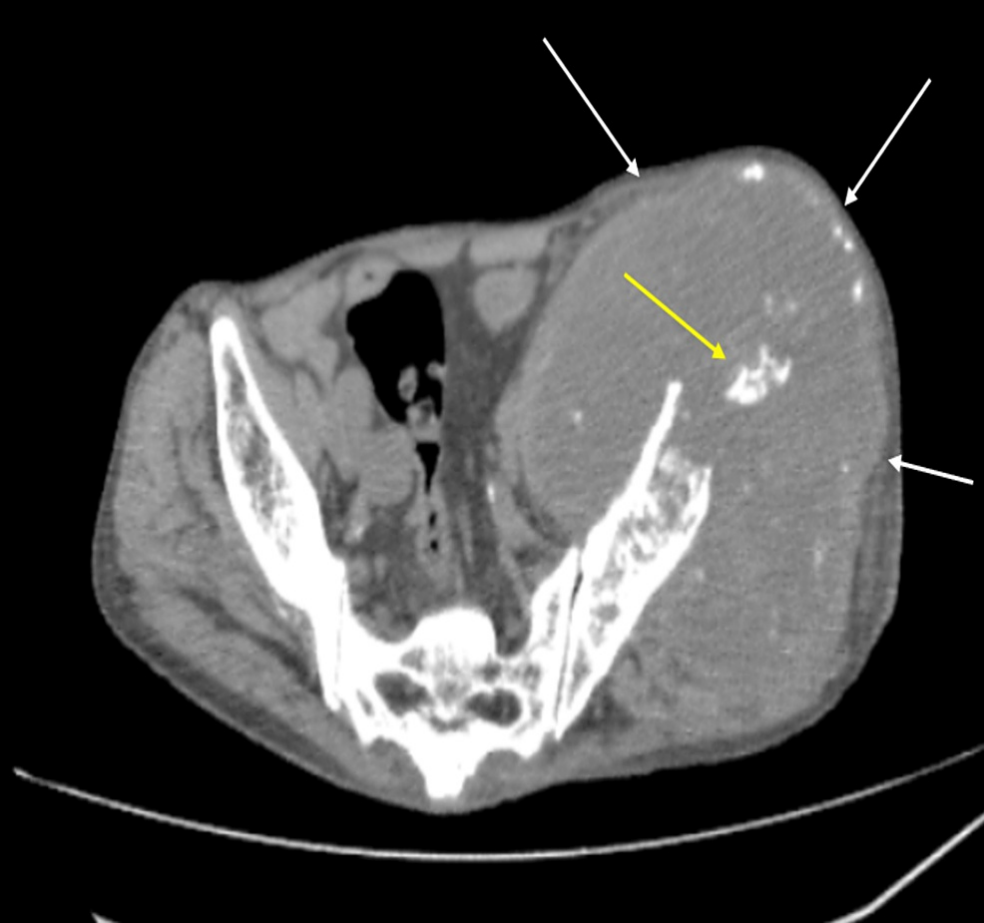
CT showing large large soft tissue mass encasing left iliac blade (white arrows). Few calcific foci are noted in central (yellow arrow) and periphery.

On CT, an ill-defined soft tissue mass measuring 18 x 13.2 x 11.5 cm encased the iliac blade on the left side, extending from the left hip to the left paraspinal muscles. There was a mixture of hyperdense and hypodense areas noted reflecting the heterogeneous nature of the tumour. Central and peripheral calcifications were noted within the tumour (Figure 3).

**Figure 4:**
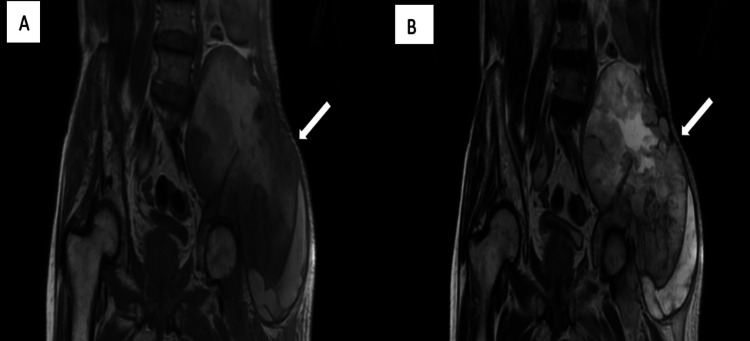
(A) Coronal T1WI image showing large soft tissue mass (white arrow) encasing the left iliac blade appearing isointense; (B) On coronal T2WI image, same mass appears hyperintense with few cystic spaces

**Figure 5:**
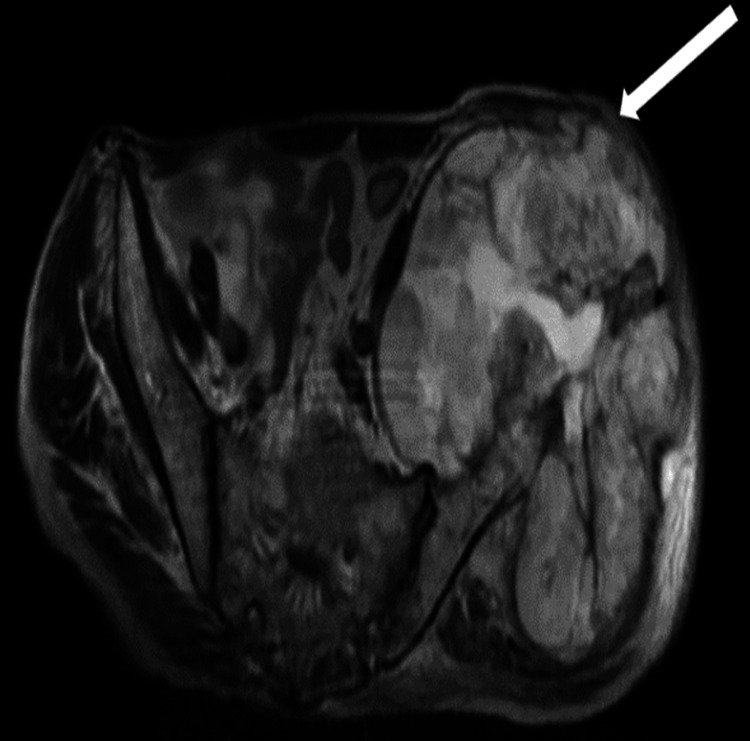
On STIR imaging hyperintensity is noted within the lesion suggestive of cystic changes. STIR: short tau inversion recovery

**Figure 6:**
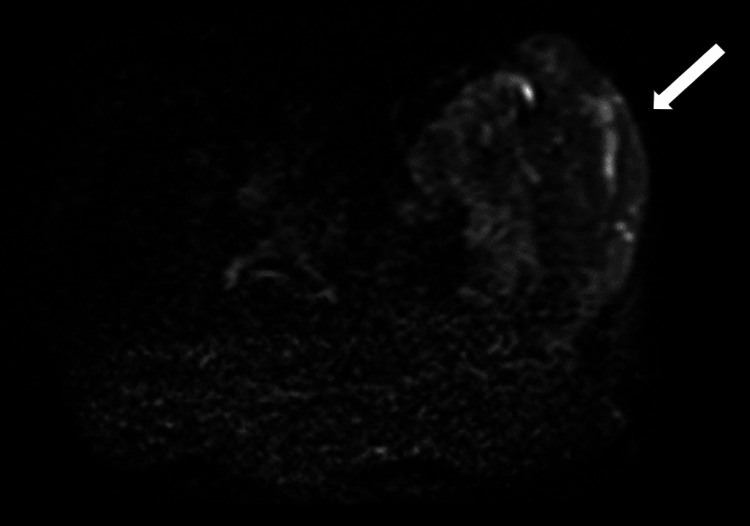
Diffusion-weighted imaging shows mild diffusion in the lesion site (white arrow).

**Figure 7:**
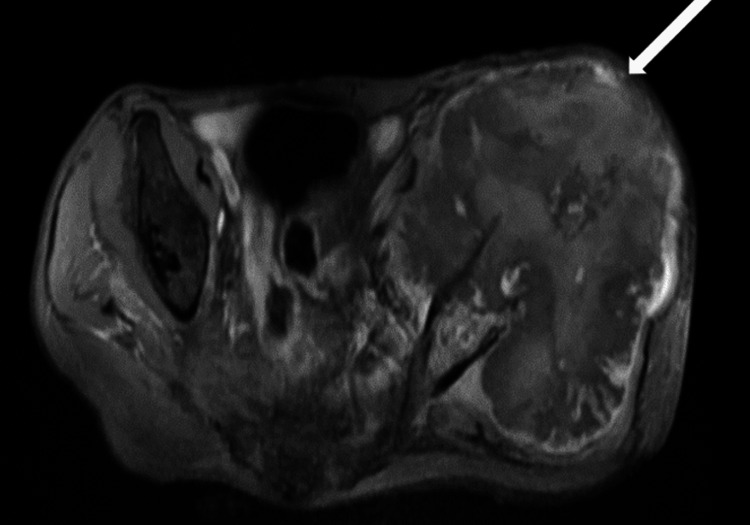
On post-contrast imaging, heterogeneous central and peripheral enhancement is noted.

**Figure 8:**
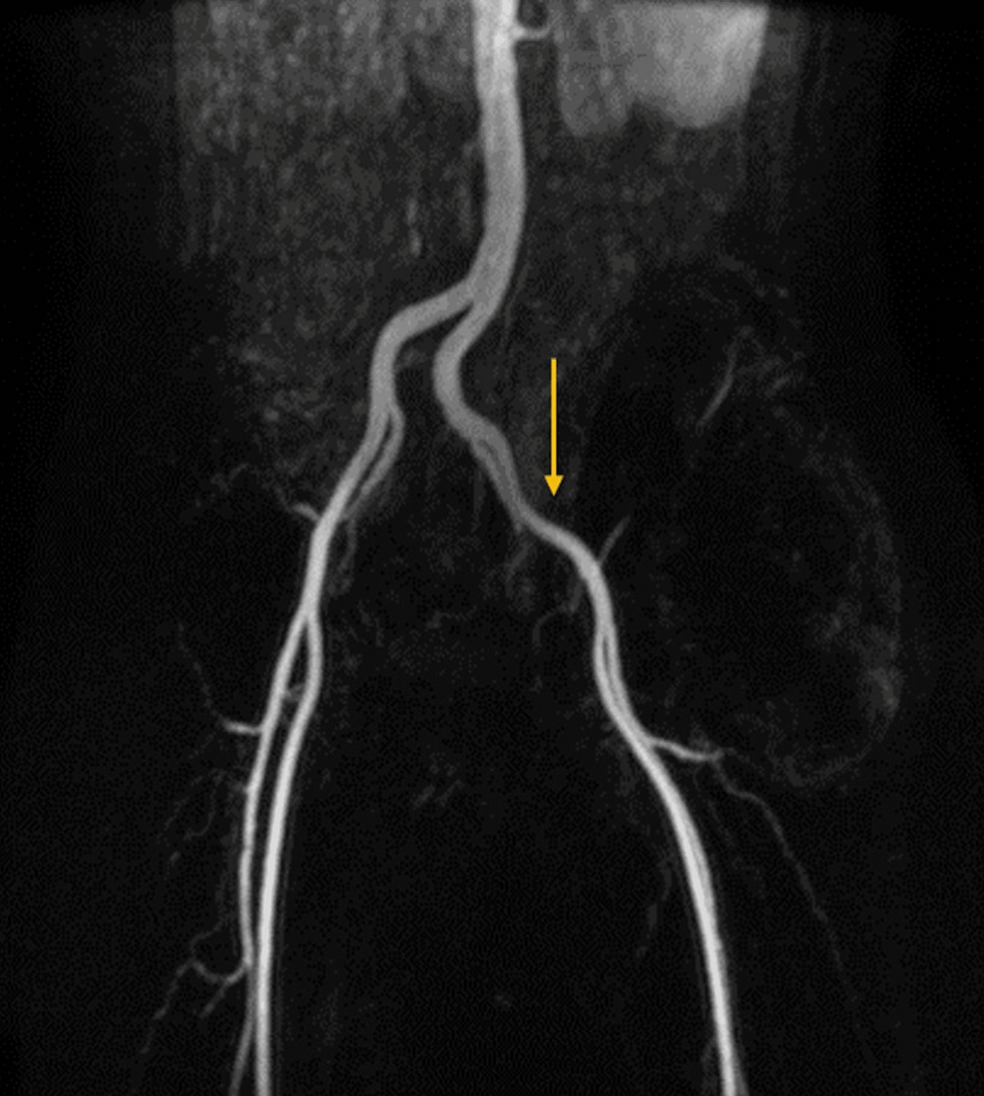
On TRICKS angio sequence, the lesion is seen to be displacing the common, external, and internal iliac artery towards the right side; however, no invasion is noted. TRICKS: Time Resolved Imaging of Contrast Kinetics

MRI revealed altered signal intensity in an 18.7 x 12.4 x 13.8 cm lesion within the left hip subcutaneous layer, extending to the para-spinal space. It appeared iso-hypointense on T1WI, mostly hyperintense on T2WI (Figure 4). It appeared hyperintense on short tau inversion recovery (STIR) with cystic spaces within (Figure 5). It shows restricted diffusion on diffusion-weighted imaging (Figure 6). It showed heterogeneous enhancement, the lesion encased the left iliac blade causing bone destruction (Figure 7). On the Time Resolved Imaging of Contrast Kinetics (TRICKS) angio sequence, the lesion is seen to be displacing the common, external and internal iliac artery towards the right side; however, no invasion is noted (Figure 8).

**Figure 9:**
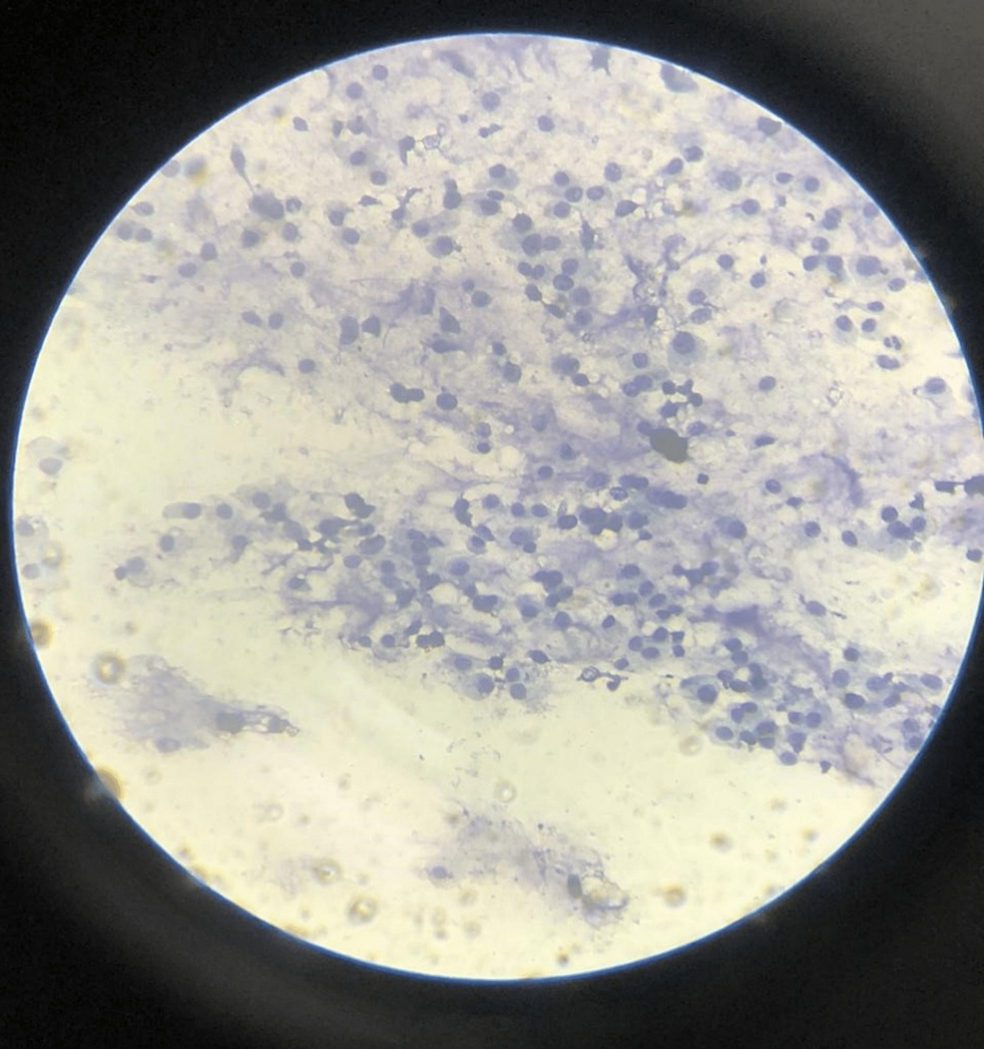
Clusters of monomorphic cells and few scattered large cells with abundant eosinophilic cytoplasm. Background shows myxoid material.

The patient underwent fine needle aspiration cytology (FNAC), which revealed clusters of monomorphic cells and a few scattered large cells. The cells were bland with abundant eosinophilic and vacuolated cytoplasm. Few cells showed mild anisonucleiosis and binucleation. The background showed myxoid material; however, mitosis and osteoclastic giant cells were not seen. It depicted low-grade chondrosarcoma (Figure 9).

## Discussion

Chondrosarcoma, a malignancy generating cartilage matrix, presents as primary (de novo) or secondary (superimposed on benign cartilaginous neoplasms). Categorized by osseous location, it encompasses central and peripheral types, the latter further divided into those arising from a preexisting osteochondroma and those developing on the bone surface (juxtacortical) [2]. In descending order of occurrence, central chondrosarcomas are commonly found in the proximal femur, iliac bone, pubic bone, sacrum, and ischial bone. In the case of peripheral chondrosarcomas, the frequency distribution is as follows: iliac bone, pubic bone and proximal femur. Moreover, these tumours possess high fragility, rendering them prone to breakage during surgical handling, thereby increasing the likelihood of tumour spillage within the surgical site. This, in turn, frequently leads to the occurrence of multiple soft tissue recurrences [3].

Conventional intramedullary chondrosarcoma, commonly termed central chondrosarcoma, constitutes the predominant primary subtype. Clinical manifestations are vague, with pain as the prevailing symptom in over 95% of cases. This pain, often gradual and worsening at night, persists for months to years prior to diagnosis. A palpable soft-tissue mass or swelling has been noted in 28-82% of individuals [2].

Plain films serve to depict and locate lesions, revealing their cartilaginous nature and aggressiveness. Central chondrosarcoma, the prevalent type, originates in the metaphysis and extends to the diaphysis. The lytic lesion displays well-defined characteristics, often with mixed lytic and sclerotic features, including characteristic ring-and-arc calcification patterns, endosteal scalloping, cortical changes, and irregular margins in high-grade cases. Calcifications within the tumour matrix vary in pattern, presence, and density. When invading soft tissue, the mass becomes palpable [4]. Ultrasound doesn’t have any specific role in bone tumours if the tumour is restricted to the intramedullary cavity and cortical destruction is absent. If the bone tumours are present with cortical destruction and involve adjacent soft tissue, colour Doppler gives an idea about soft tissue changes, cystic areas within, and vascularity. However, colour Doppler doesn’t give a clear idea about malignancy [5].

CT imaging offers excellent matrix mineralization detection, crucial for intricate anatomy or subtle cases. Distinct matrix mineralization patterns are widespread within the lesion. Three-dimensional CT imaging assists in assessing endosteal scalloping depth, showing long bone chondrosarcoma, and exhibiting significant scalloping. Lobulated endosteal scalloping and cortical response are typical in chondrosarcomas. More the soft tissue involvement, higher will be the grade on histopathology. CT with contrast showcases mild enhancement, while higher-grade lesions exhibit increased attenuation and pronounced contrast enhancement due to cellularity. MRI is highly effective for assessing marrow involvement by conventional intramedullary chondrosarcoma. T1-weighted images depict marrow replacement as low-intermediate signal intensity, revealing entrapped yellow marrow. The lobular architecture is evident, especially at the margin, and non-mineralized chondrosarcoma components exhibit high signals on T2-weighted images. Matrix mineralization, seen on MRI, appears as a low signal. Peritumoral oedema, detected through water-sensitive sequences, aids in distinguishing intramedullary chondrosarcoma from enchondroma [2]. Malignant bone tumours display heightened glycolysis and 18F-fluorodeoxyglucose (FDG) uptake. Positron emission tomography (PET)/CT's combined molecular and morphological analysis proves a potent tool for precise whole-body staging and restaging, augmenting diagnostic capabilities effectively [6].

Unlike the prevalence of chondrosarcoma in long bones, the pelvis is a common site for chondrosarcoma. Pelvic chondrosarcomas frequently affect the ileum, with a notable inclination toward the triradiate cartilage region. Due to delayed symptom onset, these lesions are often large at initial assessment. Paradoxically, though sizable, radiographs may show subtle abnormalities due to pelvic anatomy complexity [2].

There are various types of primary chondrosarcoma, including conventional intramedullary, clear cell, juxtacortical, myxoid, mesenchymal, extraskeletal, and dedifferentiated. These are differentiated by histopathologic appearance [2]. However, bone tumours like enchondroma, osteochondroma, Ewing’s sarcoma, and giant cell tumours are some differential diagnoses [3].

## Conclusions

Chondrosarcoma, a rare malignancy arising from cartilage cells, exhibits diverse presentations and complex imaging characteristics. Diagnostic modalities, including radiography, CT, and MRI, play pivotal roles in identifying its features and extent. Comprehensive understanding aids in accurate diagnosis and effective management.
